# How Spanish speakers express norms using generic person markers

**DOI:** 10.1038/s41598-022-08675-2

**Published:** 2022-03-23

**Authors:** Cristina E. Salvador, Ariana Orvell, Ethan Kross, Susan A. Gelman

**Affiliations:** 1 Department of Psychology and Neuroscience, Duke University, 417 Chapel Drive, Box 90086, Durham, NC 27708 USA; 2 Department of Psychology, Bryn Mawr College, Bryn Mawr, USA; 3 Department of Psychology, University of Michigan, Ann Arbor, USA; 4 Ross School of Business, University of Michigan, Ann Arbor, USA

**Keywords:** Psychology, Human behaviour

## Abstract

Language is one powerful vehicle for transmitting norms—a universal feature of society. In English, people use “you” generically (e.g., “You win some you lose some”) to express and interpret norms. Here, we examine how norms are conveyed and interpreted in Spanish, a language that—unlike English—has two forms of you (i.e., formal, informal), distinct generic person markers, and pro-drop, allowing for an examination of underlying conceptual tendencies in how the structure of language facilitates the transmission of norms. In Study 1a-b (N = 838) Spanish speakers used informal generic-you and the generic person marker “se” (but not formal-you) to express norms (vs. preferences). In Study 2 (N = 300), formal you, informal you, and impersonal “se” had persuasive force over personal endorsements (e.g., “I”), informing Spanish speaker’s interpretation of unfamiliar norms. Our findings add to a growing literature on how subtle linguistic shifts reflect and influence cognitive processes.

## Introduction

Norms are integral to our social worlds. They dictate behaviors that are expected, typical, and valued in a given context, allowing individuals to effectively coordinate their behavior^1,2^. Although norms can be transmitted in myriad ways (e.g., direct instruction, observation), one powerful mechanism is through language^3^. To date, the majority of empirical research on how language conveys norms has been with people whose first language is English^4^. Identifying what linguistic mechanisms are used to convey norms in other languages has the potential to shed light on whether there are common conceptual underpinnings in how languages facilitate the transmission of shared social expectations. We begin to address this question by examining how norms are expressed and interpreted in Spanish—which is one of the world’s most commonly spoken languages^5^.

## Generic person markers and norms in English

In English, norms can be explicitly conveyed with auxiliary verbs such as “should,” “ought,” or “must.” However, norms can also be expressed indirectly. One mechanism is through generic noun phrases such as “Boys don’t cry,” which convey generalizations about categories (e.g., boys) and tend to take on a normative quality^6–9^. Another indirect mechanism is *generic person markers*, which are linguistic devices that refer to people in general^10^. Generic person markers can be pronouns such as “one” or “you” (e.g., “One covers one’s mouth when one sneezes”; “You cover your mouth when you sneeze”). Unlike generic noun phrases, which express generalizations about a specific category (e.g., boys, cats, teachers), generic person markers are broader in scope because they refer to people in general. Given that norms are inherently general—providing information about how *groups* of people act or should act—generic person markers may be a particularly powerful linguistic mechanism for indirectly conveying normative information^11–13^.

The most common generic person marker in American English is the generic usage of the word “you” (hereafter generic-you). In a series of experiments, Orvell and colleagues found that adults and children used generic-you to talk about norms more than preferences^12,14^, and that people inferred a given behavior was normative when it was expressed with generic-you, compared to when it was expressed with first-person singular pronouns, such “I”^13^. These studies highlight how a subtle but ubiquitous form of speech powerfully signals norms and shapes people’s interpretation of them.

Whether these findings generalize to other languages, including Spanish, is unknown. Addressing this question is important for two reasons. First, much of the research in psychological science is conducted with samples composed of English speakers, which limits our understanding of how basic psychological processes operate among individuals in the rest of the world^15,16^. Identifying how people who speak other languages express norms—directly and indirectly—can elucidate whether there are common conceptual and linguistic underpinnings for the mechanisms that most readily convey them. Spanish provides a strong test of this idea because there are several key aspects of the language that differentiate it from English. These include multiple forms of “you”, distinct generic person markers, and pronoun drop (i.e., ‘pro-drop’, where pronouns are omitted and, in Spanish, signaled through verb conjugations and inferred from context).

The overarching aim of these studies was to test two related questions: First, are generic person markers used to express and influence the interpretation of norms in Spanish, as they are in English? Our second aim was to examine which generic person markers are most commonly used to express and influence people's interpretation of norms in Spanish. In particular, we were interested in testing whether generic-you would be used to express norms in Spanish as in English.

## Generic person markers and norms in Spanish

Data from one in-depth analysis of interviews with fourteen Spanish speakers provides initial evidence that the pronoun “you” can be used generically in Spanish^17^. Whether it is systematically used to express and influence the interpretation of norms is unknown. Based on prior work in English^12^, we hypothesized that Spanish speakers would use “you” generically to express and inform their interpretation of norms. However, unlike English (but as in many other languages), Spanish has two forms of “you”: “tú”, referred to as informal-you, and “usted”, referred to as formal-you. Whereas informal-you is typically used in contexts with equals or those of lower status (e.g., friends or students), formal-you is typically used with those higher in status (e.g., a person who is older or one’s boss). For example, a teenager speaking to their friend may ask, “¿Qué haces tú en un día de lluvia? (“What do you do on a rainy day”; informal-you pronoun tú can also be omitted), whereas a teenager asking their teacher would be more likely to use formal-you and ask, “¿Qué hace usted en un día de lluvia? (“What do you do on a rainy day” formal-you pronoun usted can also be omitted).

This feature of the language provides an opportunity to discern the underlying motivation for using generic-you to convey norms. Because informal-you assumes that the speaker and addressee are on the same level, a preference for informal generic-you could suggest an affiliative or equalizing goal, describing how behaviors apply to anyone and everyone. A preference for formal-you, on the other hand, could suggest that generic-you is functional in normative contexts because it is hierarchical and places distance between the speaker and addressee.

There are other linguistic mechanisms that might be used to express norms in Spanish. Specifically, the word “se” can be used to refer to a generic person; this usage of “se” is known as impersonal “se”. For example, “Se trabaja de Lunes a Viernes” can be roughly translated as “A person works from Monday to Friday”. When “se” is used generically, there is no grammatical agent—i.e., no one is responsible for enacting the verb. There are also other, more direct, ways of referring to generic persons in Spanish which could be used to express norms, for example, “one” (i.e., “uno”; “Uno trabaja de Lunes a Viernes”) or “people” (i.e., “personas”; “Personas trabajan de Lunes a Viernes”). Additionally, it is possible that the first-person plural pronoun “we” (i.e., “nosotros”) could be used generically in Spanish. For example, “En España, trabajamos de Lunes a Viernes” (In Spain, we work from Monday to Friday). We were interested in whether these generic person markers would preferentially be used to convey normative information.

One additional feature of Spanish that differentiates it from English is that it is a pro-drop language^18^. In pro-drop languages, subject pronouns (e.g., “I,” "you") at times do not need to be explicitly included in a sentence, but rather are understood through verb conjugations and/or context^19^. For example, a person could say either "Tú estás feliz cuando tú celebras un cumpleaños" (You are happy when you celebrate a birthday) or "Estás feliz cuando celebras un cumpleaños " ([You] are happy when [you] celebrate a birthday; with brackets indicating the pronoun that is signaled through the conjugated verb). Both sentences are understood as meaning the same thing, even though only the first includes the pronoun "you” explicitly. Whether the act of dropping the pronoun cues a more (or less) general interpretation is an open question. Moreover, it is unknown whether pro-drop is more (or less) commonly used to express a norm. A secondary aim of the present work was to test this question.

## Present research

In Studies 1a and 1b, our aims were to identify whether or not generic person markers are used to talk about norms more than preferences, as they are in English. We were further interested in identifying which generic person markers were used most often, to shed light on the potential mechanisms underlying the use of generic person markers to communicate norms. Study 1b examined the role of pro-drop in facilitating a normative (vs. specific) interpretation. In Study 2, we examined whether the most observed generic pronouns used to convey norms in Studies 1a-b had persuasive force, affecting people’s interpretation of norms. We additionally examined whether the persuasive force of some generic person markers was stronger than others. We hypothesized that Spanish speakers would rely on subtle means of referring to people in general to both discuss and interpret norms, based on the universality of generic person markers^10^, but we were unsure as to which generic person markers would be most commonly used and most persuasive.

## Study 1a

### Results

#### Specific vs. generic responses

**Figure 1:**
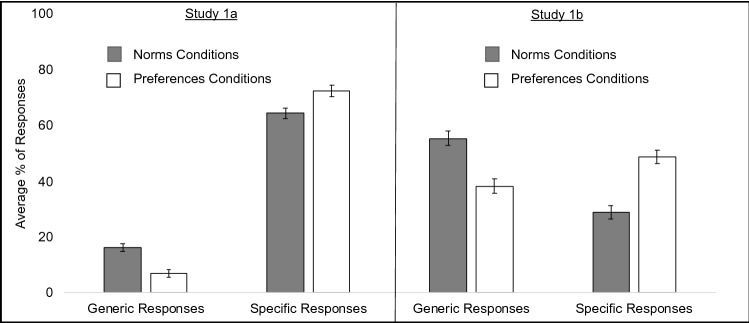
The average percent of generic and specific responses in Study 1a (left panel) and Study 1b (right panel) in the norms and preferences conditions collapsed across the question stem.

We began by testing whether participants in the Norms condition were more likely to provide responses that referred to people in general (e.g., through generic person indicators such as generic-you, generic-we, “one”), and whether participants in the Preferences condition were more likely to provide responses that conveyed a specific, first-person perspective (e.g., “I”). We omitted incomplete and ambiguous responses. To test this, we ran a mixed ANOVA where Condition (norms vs. preferences) and Question stem (formal vs. informal) were entered as between-subject factors and participants’ Responses (generic vs. specific) were entered as a within-subjects factor. Overall, people were more likely to provide specific (*M* = 68.32, *SE* = 1.39) vs. generic (*M* = 11.63, *SE* = 0.96) responses, as shown by a significant main effect of Response, *F*(1, 434) = 860.39, *p* < 0.001, *η*_*p*_^*2*^ = 0.665. This main effect was qualified by a significant Condition X Response interaction, *F*(1, 434) = 19.02, *p* < 0.001, *η*_*p*_^*2*^ = 0.042. As shown on the left panel of Fig. 1, people were more likely to provide generic responses when cued to think about norms as opposed to preferences, *F*(1, 434) = 22.02, *p* < 0.001, *η*_*p*_^*2*^ = 0.048. In contrast, people were more likely to provide specific, personal responses (i.e., using first-person singular pronouns) when cued to think about preferences as opposed to norms, *F*(1, 434) = 8.06, *p* = 0.005, *η*_*p*_^*2*^ = 0.018. This supports the idea that generic person indicators are used to convey norms in Spanish, although at lower rates than in English^12^. None of the other main effects nor interactions achieved statistical significance.

### Differences between generic person markers

**Figure 2:**
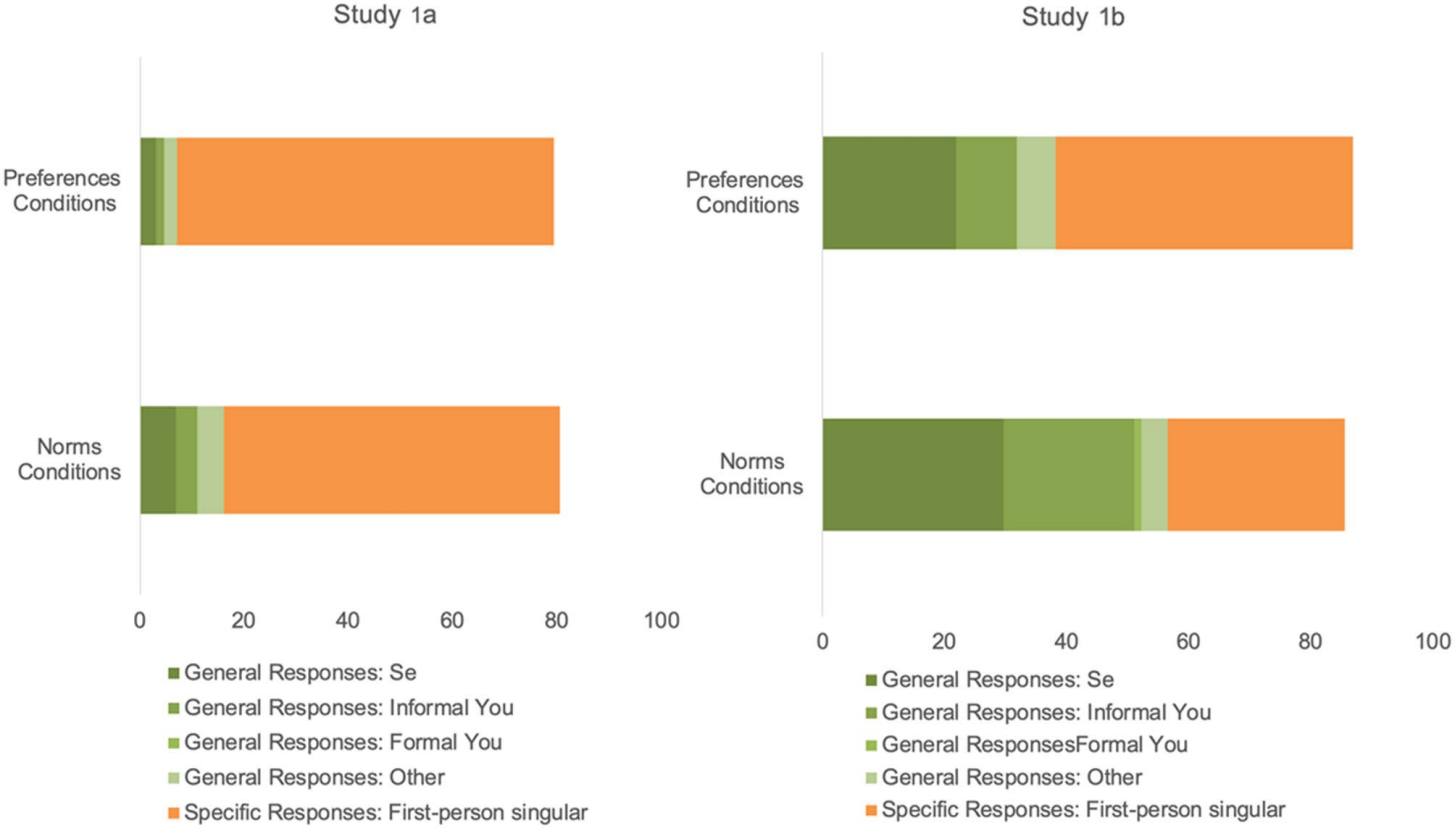
The average percent of generic and specific responses in Study 1a (left) and Study 1b (right) in the norms and preferences conditions collapsed across the question stem (i.e., informal-you and formal-you). Generic responses are separated by the generic person indicators of interest: “se”, informal-you, formal-you and other (e.g., “we”, “one”).

We next explored differences in how the three generic person markers of interest (se, tú and usted) were used *within* the norms conditions. Specifically, we tested whether the Response (se, tú, or usted) that participants provided varied based on the Question stem (formal vs. informal) they received. As shown in Fig. 2, there was a significant main effect of Response, *F*(1, 434) = 36.92, *p* < 0.001, *η*_*p*_^*2*^ = 0.15. All three generic person markers differed in how frequently they were used. Participants were most likely to respond with se (*M* = 8.92, *SE* = 1.03) followed by tú (*M* = 4.05, *SE* = 0.86) and usted (*M* = 0.06, *SE* = 0.06), *ps* < 0.001. This main effect was qualified by an interaction between Response and Question stem, *F*(1, 434) = 6.22, *p* = 0.002, *η*_*p*_^*2*^ = 0.028. When the question was framed informally (i.e., with informal-you, tú), se (*M* = 6.25, *SE* = 1.45) and tú (*M* = 4.89, *SE* = 1.21) were used significantly more than usted (*M* = 0, *SE* = 0.081), *p*s < 0.001. The former two did not differ from each other, *p* = 0.419. When the question was posed formally (i.e., with formal-you, usted), all three generic person markers differed from each other: se (*M* = 11.58, *SE* = 1.46) was used most often, followed by tú (*M* = 3.21, *SE* = 1.21) and usted (*M* = 0.115, *SE* = 0.081), *ps* < 0.001. Means and standard deviations for all generic and specific responses which we coded for are provided in Table 1.

**Table 1:** Means for the responses coded in Study 1a (top) and Study 1b (bottom). Each number represents a percent out of 100.

	Generic interpretation	Impersonal ‘Se’	Informal generic you	Formal generic you	Generic people	Generic we	Specific interpretation
Study 1a							
Norms							
Informal you	13.07 (2.11)	6.25 (1.04)	4.89 (1.39)	0.00 (0)	0.68 (0.39)	3.07 (0.79)	67.27 (2.87)
Formal you	19.15 (2.40)	11.58 (1.78)	3.21 (1.01)	0.11 (0.11)	2.18 (0.61)	2.87 (0.60)	61.47 (2.94)
Preferences							
Informal you	5.80 (1.22)	2.27 (0.65)	1.82 (0.852)	0.00 (0)	0.57 (0.38)	1.59 (0.40)	72.27 (2.62)
Formal you	8.49 (1.72)	3.90 (1.00)	1.26 (0.63)	0.00 (0)	1.26 (0.46)	3.21 (0.88)	72.25 (2.67)
Study 1b							
Norms							
Informal you	54.20 (3.38)	23.95 (2.55)	27.31 (2.99)	0.28 (0.20)	3.36 (0.82)	6.16 (1.41)	34.59 (3.43)
Formal you	56.57 (3.61)	35.39 (3.16)	15.57 (2.61)	1.86 (0.72)	6.00 (1.60)	6.43 (1.43)	23.29 (3.16)
Preferences							
Informal you	37.66 (3.53)	17.60 (2.26)	15.44 (2.58)	0.00 (0)	2.74 (0.78)	6.49 (1.48)	52.67 (3.71)
Formal you	38.75 (3.40)	26.14 (2.90)	4.26 (1.24)	0.20 (0.21)	3.50 (0.86)	7.29 (1.41)	44.68 (3.69)

Standard errors are in parentheses. The column labeled generic interpretation is the sum of the generic person indicators ‘se’, informal generic-you, formal generic-you, generic people and generic we.

### Discussion

Consistent with prior work in English^12^, Spanish speakers were more likely to use generic person markers when discussing *norms* as opposed to *preferences*. These effects are noteworthy given that the questions that participants read were identical across the norms and preferences conditions—only the context which cued people to consider “shoulds and shouldn’ts” (i.e., norms) or “likes and dislikes” (i.e., preferences) differed. To our knowledge, this is the first study to demonstrate people’s tendency to express norms with generic person markers in a language other than English, providing a common conceptual link between generic person markers and the expression of normative information.

The most commonly used generic person marker was impersonal “se”, suggesting the link between generic person markers and norms isn’t restricted to generic-you. Informal generic-you was used to express norms with relative frequency, whereas formal generic-you was not used even when it was provided in the question. Thus, our data suggest when “you” is used generically in Spanish, it is done with a form of “you” that assumes equality between speaker and listener (i.e., informal-you), rather than a form of “you” that distances speaker from listener (i.e., formal-you).

However, the rates of generic person markers were markedly lower than those observed with English speakers, 11% as opposed to the approximately 35% observed by Orvell et al. (2017). One explanation for the low base rates in the norms conditions may be that the sentence frames in Study 1a included subject pronouns (e.g., “tú” in “Como celebras tú un cumpleaños?”). Given that Spanish is a pro-drop language where subject pronouns are often dropped, it is possible that by including the subject pronouns for informal-you and formal-you, the pronouns were made more salient, increasing the likelihood that participants would interpret the “you” as specific (i.e., as referring to them) rather than as referring to people in general. This may be akin to an English speaker placing stress on the word “you” (“How do ***you cook a turkey?***”), to communicate that the “you” refers to the addressee and is not generic^20,21^. Study 1b examined this possibility by replicating Study 1a, but without including subject pronouns in the question stems.

## Study 1b

### Results

#### Specific vs. generic responses

As in Study 1a, we ran a mixed ANOVA where Condition (norms vs. preferences) and Question stem (formal vs. informal) were entered as between-subject factors and Response (generic vs. specific) was entered as a within-subjects factor. There was a significant main effect of Response, *F*(1, 391) = 5.98, *p* = 0.015, *η*_*p*_^*2*^ = 0.015, which showed that people were more likely to provide responses that signaled a generic (*M* = 46.80, *SE* = 1.74) as opposed to specific (*M* = 38.81, *SE* = 1.75) interpretation. This is the opposite pattern to that observed in Study 1a, suggesting that the mere act of dropping the pronoun led to a 3.5-fold increase in people’s tendency to provide a generic response.

As in Study 1a, this main effect was qualified by a significant Condition X Response interaction, *F*(1, 391) = 31.94, *p* < 0.001, *η*_*p*_^*2*^ = 0.076. As illustrated in Fig. 1 (right panel), people were more likely to provide responses that signaled a generic interpretation in response to questions about norms as opposed to preferences, *F*(1, 391) = 24.29, *p* < 0.001, *η*_*p*_^*2*^ = 0.058. In contrast, people were more likely to provide specific, personal responses (i.e., using first-person singular pronouns) when cued to think about preferences as opposed to norms, *F*(1, 391) = 31.76, *p* < 0.001, *η*_*p*_^*2*^ = 0.075. Means for all types of responses are provided in Table 1.

### Differences between generic person markers

We next explored differences in how the three generic person markers of interest (se, tú, and usted) were used *within* the norms conditions. We again tested whether the Response (se, tú, or usted) that participants provided varied based on the Question stem (formal vs. informal) they received. As shown in Fig. 2, there was a significant main effect of the Response, *F*(2, 324.31) = 78.92, *p* < 0.001, *η*_*p*_^*2*^ = 0.28, which showed that se (*M* = 29.62, *SE* = 2.03) was used significantly more than tú (*M* = 21.44, *SE* = 1.99), *p* = 0.005 and usted (*M* = 1.07, *SE* = 0.37), *ps* < 0.001.This effect was qualified by a Response x Question stem interaction, *F*(2, 324.31) = 12.25, *p* < 0.001, *η*_*p*_^*2*^ = 0.058. As in Study 1a, when the question was phrased informally (i.e., the verbs were conjugated to signal informal you, tú), se (*M* = 23.95, *SE* = 2.85) and tú (*M* = 27.31, *SE* = 2.79) were used significantly more than usted (*M* = 0.28, *SE* = 0.52), *p*s < 0.001. The former two did not differ from each other, *p* = 0.403. When the question was posed formally (i.e., the verbs were conjugated to signal formal-you, usted), se (*M* = 35.39, *SE* = 2.88) was the most frequently used, followed by tú (*M* = 15.57, *SE* = 2.82) and usted (*M* = 1.86, *SE* = 0.528), *p*s < 0.001.

### Discussion

Replicating Study 1a, we found that participants were more likely to use generic person markers when discussing *norms* as opposed to *preferences*. Notably, framing the questions using pro-drop led to an almost 3.5-fold increase in the usage of generic person markers in the norms condition, yielding rates similar to what has been observed in prior work, with English speakers. However, unlike prior work with English speakers, the usage of generic person markers was also high in the preferences condition (though importantly, not as high as in the norms condition). This suggests that pronoun drop may overwhelmingly cue a general interpretation that extends even to contexts where a specific interpretation is typically more common (i.e., in the case of preferences).

The findings from Studies 1a-1b identified “se” and informal-you as the most used generic person markers used to express norms. However, it is unknown whether these generic person markers also influence how people interpret information, carrying persuasive force. We examined this question in Study 2.

## Study 2

In Study 1, we found that Spanish speakers used generic person markers to talk about *norms* as opposed to *preferences*. We suggest this occurs because these phrases refer to people in general, and thus are an appropriate way to communicate normative information, which applies broadly. In Study 2, we examined whether these generic person markers also have persuasive value, affecting how people interpret unfamiliar norms. We focused on the persuasive force of the impersonal markers “se” and informal-you, given that they were frequently used to express norms in Study 1. We included formal-you to be comprehensive, even though it was less commonly generated to express norms.

### Results

#### Persuasive force of formal-you and informal-you

Following prior work (Orvell et al., 2019), we calculated the percent of trials in which participants selected the behavior described with a generic person marker. We then conducted one-sample t-tests against chance (i.e., 50%) for each of the three between subject contrasts (i.e., impersonal “se” + “I”; formal-you + “I”; informal-you + “I”) that participants were randomly assigned to.

Participants selected behaviors described with impersonal “se” (*M* = 78.00, *SD* = 31.03) and formal-you (*M* = 61.27, *SD* = 34.58) as representing the correct way to do things significantly above chance, *t*(99) = 9.02, *p* < 0.001, *d* = 0.90 and *t*(101) = 3.29, *p* = 0.001, *d* = 0.33, for impersonal “se” and formal-you, respectively. Surprisingly, behaviors described with informal-you were not selected significantly above chance (*M* = 55.45, *SD* = 36.84), *t*(100) = 1.49, *p* = 0.141, d = 0.15.

#### Difference in persuasive force among generic person indicators

To test whether the 3 types of generic person indicators (informal-you, formal-you, and se) differed in their persuasive force, we conducted an ANOVA. There was a significant main effect of Condition, *F*(2, 300) = 11.74, *p* < 0.001, *η*_*p*_^*2*^ = 0.073; impersonal “se” was more persuasive than both formal-you and informal-you, *p*s < 0.001. Formal-you and informal-you did not significantly differ from each other, *p* = 0.226.

#### Secondary analyses: probing participants’ interpretations

Despite our efforts to ensure that participants would interpret the pronouns used in the trials as generic (i.e., referring to people in general), our debriefing data revealed there was variability in how participants interpreted informal-you and formal-you. Impersonal “se” was always interpreted as referring to people in general; however, 38% of participants in the informal-you condition and 20% of participants in the formal-you condition reported that they interpreted “you” as referring to “only [themselves] and no one else” (rather than to people in general).

Given this, we re-ran the analyses described above, but only included people who interpreted the pronouns generically. As expected, these analyses now revealed that participants endorsed behaviors described with informal-you (vs. “I”) as representing the correct way to do things significantly above chance (*M* = 66.94, *SD* = 37.54, *t*(61) = 3.55, *p* = 0.001, *d* = 0.45). As before, behaviors described with formal-you (vs. “I”) were also viewed as representing the correct way to do things significantly above chance (*M* = 67.81, *SD* = 34.26, *t*(79) = 4.65, *p* < 0.001, *d* = 0.52). Despite being perceived as more persuasive than I, both forms of generic-you varied in their persuasiveness, as indicated by a marginal effect of Condition, *F*(2, 238) = 3.00, *p* = 0.051, *η*_*p*_^*2*^ = 0.025. Informal and formal-you were similarly persuasive, *p* = 0.879. However, both were significantly less persuasive than impersonal “se”, *ps* < 0.041.

For comprehensiveness, we re-ran the same set of analyses, but now restricted the sample to participants who reported that informal-you and formal-you were being used to refer to them (i.e., specifically). Interestingly, these participants perceived informal-you (*M* = 37.50, *SD* = 27.72) and formal-you (*M* = 37.50, *SD* = 25.00) as significantly *less* persuasive than I, *t*(37) = -2.78, *p* = 0.009, *d* = 0.45 and *t*(19) = -2.24, *p* = 0.038, *d* = 0.50, respectively. This highlights that a generic interpretation is the essential ingredient guiding people’s interpretation of norms.

### Discussion

Study 2 provided support for the hypothesis that impersonal “se”, formal-you (usted), and informal-you (tú) have persuasive force, affecting how people interpret unfamiliar norms. This is particularly noteworthy because in all cases the generic person markers were contrasted with personal endorsements (i.e., using “I”), which prior work suggests can serve as powerful routes to persuasion^22^.

Of the three generic person indicators tested, impersonal “se” was consistently interpreted as the most persuasive. Formal-you (usted) and informal-you (tú) were only more persuasive than “I” when they were interpreted as referring people in general. When “you” was interpreted as referring specifically to the self, the opposite pattern emerged: “I” was seen as more persuasive than both specific formal- and informal-you. These findings suggest that a generic interpretation has persuasive force above and beyond a personal endorsement.

All statements in Study 2 included the pronoun, which likely explains the relatively high rates of specific (i.e., personal) interpretations for the informal-you and formal-you conditions. We included the pronouns in part because without the pronoun, the “se” and “usted” conditions would be indistinguishable. Nonetheless, future work is needed to test the possibility that rates of persuasion would be even higher if pronoun drop were used in the sentence frames.

## General discussion

The present work expands emerging research on the linguistic signals of norms from English to Spanish. By studying Spanish we were able to gain additional insights that could not be obtained by studying English alone. Studies 1a-b revealed that informal, but not formal-you was used to express norms, suggesting that the underlying motivation for the use of generic-you in Spanish is affiliative, as opposed to distancing. We also found evidence that impersonal “se” may be the most commonly used generic person marker in Spanish. Finally, pro-drop increased the likelihood that a statement was interpreted generically, showing that the inclusion of the pronoun itself might prompt a more specific interpretation. These findings provide some initial evidence for the possibility that pronoun-drop can be used to signal generic information. In Study 2, we found that both informal-you and formal-you have persuasive force, when they were interpreted as referring to people in general. Impersonal “se”, however, was the most persuasive generic person marker.

There are many possible ways to signal a general statement, and not all languages use “you” generically. However, “you” has been documented as taking on generic meaning in multiple unrelated languages (including Mandinka, Duna, and Dutch^23–25^), raising the question of why this might be. It is possible that “you” acquired a generic meaning precisely because it is often used to refer to a specific addressee(s). Generic-you broadens the scope of “you” to refer to people in general, but by using the same word used to refer to a specific person. Thus, it may be particularly powerful as a means to pique the addressee’s attention in ways that other generic pronouns do not^26^.

In Studies 1a-b people’s use of generic-you to express norms was largely restricted to informal-you (tú) and nearly absent with formal-you (usted). Although, formal-you still had persuasive force, affecting people’s interpretation of norms in Study 2. The preferred use of informal generic-you to express norms suggests that generic-you may be serving a more equalizing rather than distancing function. Thus, the generic usage of informal-you could promote the idea that the speaker is expressing norms to those of similar status. Conversely, the generic usage of formal-you might serve the function of promoting psychological distance or cueing a stricter context, where norms may be more defined (i.e., tight; Gelfand et al., 2006). It’s also possible that because formal-you is used to mark a particular social dynamic and signals hierarchy, respect, or status among two parties, it is less likely to take on a generic meaning.

Although “you” was used generically in Spanish, across both studies, the normative effects were strongest for “se”, a linguistic indicator that has no English equivalent. This finding is consistent with a prior work by Posio (2016), which did not look at norms, specifically. One potential explanation for the strength of “se” as a generic person indicator is that it is unambiguously generic. For example, in Study 2, 100% of people in the impersonal “se” condition interpreted “se” as referring to people in general (exclusively or inclusive of the self), where informal- and formal-you were interpreted specifically by some participants. Thus, it is possible that impersonal “se” may be the clearest linguistic signal in Spanish that a particular idea generalizes beyond a particular person, time, or place. This unambiguously generic quality may explain why it appears to be the preferred mechanism for expressing and influencing the perception of norms in Spanish.

## Considerations and future directions

Our studies recruited Spanish speakers from a variety of different countries. Although this allowed us to collect data from a variety of participants, also demonstrating the generalizability of this phenomenon, there are subtle, and not so subtle, differences in how Spanish is spoken across countries^27^. For example, the stimuli used in Study 1a place the pronouns before the verb (e.g., ¿Como celebras tú un cumpleaños?); however, Caribbean Spanish-speakers often place the pronoun before the verb (e.g., ¿Como tú celebras un cumpleaños?). Thus, it is possible that the constructions we used in Study 1a may have been less natural for some participants. As a more substantive grammatical difference, some South American countries use the mechansism “vos” as a replacement for informal-you^28^. Although we found no evidence that “vos” was used generically in our samples (Study 1a or 1b), which did include some South American participants, future work can directly test whether “vos” is used as a generic pronoun, for example, by designing stimuli that use this form (e.g., “¿Como celebráis vos un cumpleaños?”) In sum, future work should systematically examine whether there are differences in how generic person markers are used across Spanish-speaking countries, attending to subtleties in dialect.

Another direction for future research is to examine whether certain characteristics of a person, situation or culture may increase the likelihood for generic person markers to be used. For example, recent work shows that when a person is motivated to relate to others in a context where norms are strict (i.e., tight), they become more attuned to norms^29^. It is possible that these and other factors may influence how much generic person indicators are used to signal norms, or the extent to which people rely on them to discern what is normatively correct. Nonetheless, the present work pushes the literature one clear step forward by showing that generic language is used to express and influence the interpretation of norms in Spanish. These findings contribute to a growing literature on the bidirectional influence of language on social meaning.

## Study 1a

### Method

#### Participants

An a priori power analysis was conducted on Orvell et al.’s (2017) Experiment 3. The required sample size for each condition was 46 participants. Since the study was being conducted in Spanish for the first time and the effect size was unknown, we elected to maintain the original target sample size of 100 participants per condition. Since there is often some attrition in online studies^30^, we aimed to collect data from an additional 50 participants (i.e., a sample of 450 participants). Participants were recruited from Prolific Academic. The study was available to individuals who previously indicated to Prolific that their first language was Spanish and who indicated that they did not have any speaking or reading difficulties. 451 Spanish-as-first-language speakers (265 male, 167 female,19 not reported) who varied from 18 to 63 years of age (*M* = 30.10, *SD* = 9.24) took part in the study. Participants resided in 14 different countries with the majority in Spain (159), Mexico (174), and Chile (26). 30 participants reported living in other countries in North America (e.g., United States) and 45 in other countries in Europe (e.g., Italy). The majority of participants (414) were multi-lingual (i.e., reported speaking at least one other language in addition to Spanish) and 20 spoke only Spanish. 17 participants did not report their country or language background. Eight were excluded prior to analyses for incomplete responses and 5 for writing their answers in a language other than Spanish. All studies were approved by the University of Michigan Institutional Review board and all relevant guidelines and regulations were followed. Informed consent was obtained from all participants. All participants were compensated at Prolific’s minimum of $6.50/h (which corresponded to $1.63 based on the survey length).

#### Design

We used a 2 × 2 between-subjects design; participants were randomly assigned to answer questions while cued to think about either Norms or Preferences, with the questions framed using either informal-you (tú) or formal-you (usted). Participants completed eight trials. These studies were not formally pre-registered.

#### Procedure

All task materials (including instructions) were presented in Spanish. Spanish speakers were recruited through Prolific Academic for a study on everyday language use. They agreed to write in full sentences after providing informed consent. All materials from Orvell et al.’s (2017) Experiment 3 were translated to Spanish and back-translated by a professional translator and two native Spanish speakers to ensure equivalence in meaning. Participants were randomly assigned to one of four conditions: informal-you (tú) + Norms (*N* = 112), informal-you (tú) + Preferences (*N* = 112), formal-you (usted) + Norms (*N* = 110), or formal-you (usted) + Preferences (*N* = 109).

Following prior work, all participants were asked to imagine that they had to describe to an alien what life was like on Earth^12^. In the Norms conditions, participants were told that the alien wanted to learn about norms, such as what should and should not be done on earth. In the Preferences conditions, participants were told that the alien wanted to learn about preferences, such as likes and dislikes on earth. Then, participants were asked to respond to eight randomly presented questions about common everyday behaviors (e.g., How do you celebrate a birthday?) using full sentences (see Table 2 for full stimuli). In Spanish, the same sentence could be said in two ways, with informal-you and formal-you (i.e., ¿Cómo celebras **tú** un cumpleaños? and ¿Cómo celebra **usted** un cumpleaños?, respectively; emphases added here). As described above, which form of “you” was used was varied across participants. Crucially—and by design—across all conditions, participants could interpret the “you” in the questions as referring to people in general or to them, specifically.

**Table 2:** Stimuli used in studies 1a-b.

Informal-you (Tú) Conditions	Formal-you (Usted) Conditions	English Translation
¿Cuándo te acuestas tú?	¿ Cuándo se acuesta usted?	When do you go to bed?
¿Cuándo desayunas [tú]?	¿Cuándo desayuna [usted]?	When do you eat breakfast?
¿Qué haces [tú] en un día de lluvia?	¿Qué hace [usted] en un día de lluvia?	What do you do on a rainy day?
¿Qué haces [tú] en la piscina?	¿Qué hace [usted] en la piscina?	What do you do in the pool?
¿Cómo cocinas [tú] un pollo?	¿Cómo cocina [usted] un pollo?	How do you cook a chicken?
¿Cómo celebras [tú] un cumpleaños?	¿Cómo celebra [usted] un cumpleaños?	How do you celebrate a birthday?
¿Dónde vas [tú] a comprar comida?	¿Dónde va [usted] a comprar comida?	Where do you buy groceries?
¿Dónde vas tú para relajarte?*	¿Dónde va usted para relajarse?*	Where do you go to relax?*

In Studies 1a-b, in the “Norms” conditions, all questions were prefaced by: “Por favor ayud al extraterrestre a aprender **lo que está permitido y lo que está prohibido** aquí en la Tierra.” (“Help the alien to learn what should and shouldn’t be done here on Earth.”) In the “Preferences” conditions, all questions were prefaced by: “Por favor ayude al extraterrestre a aprender **lo que es gustado y lo que es disgustado** aquí en la Tierra.” (“Help the alien to learn what is liked and disliked here on Earth”). In Study 1b, the verb “acostar” was replaced with “dormir,” and the final trial was not included (see “Procedure”).

After answering all questions, participants were given a manipulation check, where they were asked to indicate what they had to tell the alien about (“shoulds and should nots”, “likes and dislikes”, or “don’t know”). Participants answered some exploratory questionnaires and completed standard demographic information. All the study information (from the consent to debriefing) was written in informal-you in the informal-you condition, and formal-you in the formal-you condition. All materials, data, and code for the present article are available at: https://osf.io/3hdsc/?view_only=acb71dc7810643119a79d582c5940314.

#### Coding

Of interest was whether participants responded to the questions with linguistic devices that referred to people in general (signaling a generic interpretation) or with first-person pronouns (signaling a specific interpretation). For example, a person cued to think about norms and asked, “How do you celebrate a birthday?” could answer either “*I* eat cake” or “*You/People/One* eat(s) cake.” The former reflects a specific interpretation whereas the latter reflects a general interpretation.

Two independent condition-blind coders coded participants’ responses. One spoke Spanish as their first language, whereas the other was a university student majoring in Spanish. As shown in Table 3, responses were coded as reflecting either a specific (i.e., first-person personal) or a general perspective. When responses omitted a pronoun but included a verb conjugation that allowed for an inference about what pronoun was implied, they were coded correspondingly as either “personal” or “generic.” More specifically, responses were coded into one of four categories: (1) specific interpretation (i.e., indexed through use of a first-person singular pronoun such as “yo” meaning “I”, or through use of a verb conjugation that indicated first-person singular), (2) generic interpretation (which could include formal-you, informal-you, generic-we—either indexed through a noun/pronoun or through the verb conjugation, using pro-drop), impersonal “se,” people, or one, (3) incomplete (i.e., responses that were incomplete sentences with no subject specified), or (4) ambiguous responses (i.e., responses where, due to pronoun drop and the tense of the verb conjugations, it was ambiguous whether the response reflected a specific or a general interpretation). For example, in Spanish, “Cocina con sal” could refer to a specific person (e.g., he/she) or a generic person.

**Table 3:** Example interpretations and coding for the question “Como celebras/celebra [tú/usted] un cumpleaños?” (“How do you celebrate a birthday?”).

Response	Specific	Generic				Other		
Linguistic indicator	First-person singular	Informal-you Formal-you		Impersonal “se”	Generic-we	Other/Ambiguous		Incomplete Sentence
Spanish								
No pro-drop	Yo como pastel	Tú comes pastel	Usted come pastel	Se come pastel	Nosotros comemos pastel	La gente come pastel	Come pastel	Pastel
Pro-drop	Como pastel	Comes pastel	Coded as ambiguous	Comerse pastel	Comemos pastel	Coded as ambiguous	Come pastel	N/A
English translation	I eat cake	You eat cake	You eat cake	One/You/They eat cake	We eat cake	People eat cake	Eat cake	Cake

If participants used more than one of the indicators that we coded for in a single response, it was coded as containing both. Coders practiced on 90 trials, then they were then given the same 20% of the data, which was used to calculate interrater reliability. Reliabilities were k = 0.86 for generic, k = 0.93 for specific, k = 0.47 for ambiguous, and k = 0.71 for incomplete interpretations. Two expert coders resolved the discrepancies. The remaining 80% of the data was then split equally among the two coders.

## Study 1b

### Method

#### Participants

We set the same target sample size as Study 1a, 100 participants per condition. Prior to participation, participants were screened to be Spanish speakers (as their first language) without any speaking or reading difficulties. Participants from Study 1a were ineligible to participate in this study. 398 Spanish speakers (256 male, 137 female, 2 other) who varied from 18 to 64 years of age (*M* = 28.68, *SD* = 9.07) took part in the study. 3 participants were excluded prior to analyses for answering in a language other than Spanish. Participants resided in 15 different countries with the majority in Spain (155), Mexico (126), and Chile (63). 43 reported living in a European country (e.g., UK), 7 in North America (e.g., United States), and 1 in Japan. The majority of participants (383) were multi-lingual (i.e., reported speaking at least one other language aside from Spanish) and 12 spoke only Spanish. All participants were compensated at Prolific’s minimum of $6.50/h (which corresponded to $1 based on the survey length).

#### Design

We used the same design as Study 1a. Participants were randomly assigned to answer questions while cued to think about either Norms or Preferences, with the questions framed using either informal-you (tú) or formal-you (usted) but *without* the pronouns in the question stems (i.e., using pro-drop constructions; e.g., “*Como celebras/celebra un cumpleaños*?”). Since one of the questions from Study 1a could not have the pronoun dropped, we excluded that question from the present study and had participants complete seven rather than eight trials (materials available on OSF).

#### Procedure

The procedure was identical to Study 1a. Participants were randomly assigned to one of four conditions: informal-you (tú) + Norms (N = 102), informal-you (tú) + Preferences (N = 99), formal-you (usted) + Norms (N = 100), or formal-you (usted) + Preferences (N = 94).

#### Coding

Table 3 shows the coding categories used in the present study. Two independent condition-blind coders coded 10% of the data, which was used to calculate interrater reliability (alpha). One coder was a native Spanish-speaker, whereas the other was a fluent Spanish-speaker. Kappa reliabilities for the interpretations and subjects were 0.831 and 0.621, respectively. The remaining data was coded by one of the coders independently.

## Study 2

### Method

#### Participants

We followed prior work by Orvell and colleagues (2019) and set a target sample size of 100 participants per condition. Prior to participation, we screened participants so that only first-language Spanish speakers without any speaking or reading difficulties took our survey. Participants who took part in Study 1 were also ineligible. 306 Spanish speakers took our survey. Three did not complete the critical dependent variables. This left us with 303 participants (166 male, 129 female, 3 other, and 5 who did not answer) who varied from 18 to 71 years of age (*M* = 29.95, *SD* = 9.60). The 5 participants who did not answer the question about gender also failed to answer the rest of the demographic questions. Participants resided in 13 different countries with the majority coming from Spain (125), Mexico (101), and Chile (24). 18 participants reported living in other countries in North America (e.g., United States) and 30 in other countries in Europe (e.g., Austria). The majority of participants (287) were multi-lingual and reported speaking at least one other language aside from Spanish, whereas 11 spoke only Spanish. All participants were compensated at Prolific’s minimum of $6.50/h (which corresponded to $0.80 based on the survey length).

#### Procedure

Participants were recruited through Prolific Academic for a study on everyday language use. All materials from Orvell et al. (2019) Experiment 5a were translated into Spanish and back-translated by native Spanish speakers. Participants were randomly assigned to one of three conditions: informal-you (tú) vs. “I” (yo) (N = 101), formal-you (usted) vs. “I” (yo) (N = 102), and impersonal “se” vs. “I” (yo) (N = 100). After providing informed consent, participants were asked to complete the survey in an environment free of distractions. They were asked to imagine that they were in a foreign land where they would encounter objects they had never heard of before. Their task was to learn what to do with them. To highlight a contrast between norms and preferences, participants were told some of the people they would hear from were rule followers, who do things as they should, whereas others do things in their own way, the way they liked to. Participants were then told about 4 novel objects, the names of which were selected from a list of Spanish pseudo words^31^. Participants read about what two people had to say about the novel objects. One of the individuals spoke about what they did with ‘I’ and another with generic language (i.e., informal-you, formal-you, or impersonal “se”, depending on the condition). The full set of questions can be found on the OSF site.

After each trial, participants selected which way they thought was the correct way to do things. Between subjects, we counterbalanced whether actions, divided into two sets (set “A” and set “B”) were paired with the pronouns “tú/usted/se” or “I”, yielding four orders (see Table 4 for a list of object labels and actions associated with each object). Trials were randomized within each order. To determine whether participants interpreted the pronoun in the statements generically (as we intended) or specifically (i.e., referring to them, specifically) participants were asked to report who the pronoun in a given trial referred to, after they completed all four trials. They were able to select whether the pronoun referred to people in general, to them and people in general, or just them. We additionally probed participants’ intuitions about what they thought the purpose of the study was and how they determined the correct action. Then they filled out demographic questionnaires. As in Studies 1a and 1b, all the study information (from the consent to debriefing) was written in informal-you in the informal-you condition, and formal-you in the formal-you and impersonal “se” conditions. We used formal-you in the impersonal “se” condition, because “se” was most often used in Study 1 in the formal-you condition.

**Table 4:** Stimuli used in study 2.

Novel object	Action A		Action B	
	Spanish	English translation	Spanish	English translation
Dultas	Exponer en la sala	Display in the living room	Exponer en el comedor	Display in the dining room
Sancos	Comer por el desayuno	Eat for breakfast	Comer por el almuerzo	Eat for lunch
Rincos	Compartir con amigos	Share with Friends	Compartir con familia	Share with family
Conmes	Poner encima de sombrero	Put on top of hat	Poner encima de zapatos	Put on top of shoes

Table displays the novel objects and actions with them which were presented to participants. Note the sentences above are presented in the infinitive; between-subjects, participants were randomly assigned to receive contrasts between: informal-you (tú) vs. “I” (yo); formal-you (usted) vs. “I” (yo); and impersonal “se” vs. “I” (yo) and stimuli were conjugated accordingly. We additionally counterbalanced which actions (“A” vs. “B”) were paired with the pronouns “tú/usted/se” or “I”.

## Data Availability

The materials, data and code for the present paper is made available at: https://osf.io/3hdsc/?view_only=acb71dc7810643119a79d582c5940314.
